# The Spanish version of the Childbirth Experience Questionnaire (CEQ-E): reliability and validity assessment

**DOI:** 10.1186/s12884-016-1100-z

**Published:** 2016-11-24

**Authors:** F. J. Soriano-Vidal, A. Oliver-Roig, J. Cabrero-García, N. Congost-Maestre, A. Dencker, M. Richart-Martínez

**Affiliations:** 1 Department of Nursing, University of Alicante, Cta. San Vicente del Raspeig s/n, 03690 San Vicente del Raspeig, Alicante Spain; 2 Department of Nursing, Universidad Católica de Valencia “San Vicente Mártir”, Valencia, Spain; 3 Obstetrics and Gynaecology, Xàtiva-Oninyent Health Department, Xativa, Valencia Spain; 4 Department of English Studies, University of Alicante, San Vicente del Raspeig, Alicante Spain; 5 University of Gothenburg Centre for Person-Centred Care (GPCC), Sahlgrenska Academy, University of Gothenburg, Gothenburg, Sweden

**Keywords:** Childbirth experience questionnaire, Construct validity, Birth satisfaction, Patient satisfaction, Postpartum period, Reliability

## Abstract

**Background:**

The Childbirth Experience Questionnaire (CEQ) was originally designed to study women’s perceptions of labour and birth. The main objective of our study was to adapt the CEQ to the Spanish context and determine its psychometric properties. This would provide an opportunity to evaluate women’s experiences in order to improve evidence in the Spanish context as recommended by national guidelines.

**Methods:**

The CEQ was translated into Spanish using a standard forward and back translation method (CEQ-E). A convenience sample of 364 women was recruited from 3 Spanish hospitals; all participants were able to read and write in Spanish. Mothers with high risk pregnancies or preterm deliveries were excluded from the study. A self-administered questionnaire on sociodemographic variables was completed by participants before discharge. Data on childbirth variables were obtained from maternity records. Between 1 and 3 months postpartum a postal CEQ-E questionnaire was sent.

The CEQ-E structure was examined by a confirmatory factor analysis of polychoric correlations using a diagonally weighted least squares estimator. Reliability was assessed using Cronbach’s alpha. Construct validity was conducted by testing differences in CEQ-E scores between known-groups (to differ on key variables).

**Results:**

226 (62.1%) of the recruited participants completed the postal questionnaire. The CEQ-E factor structure was similar to the original one. The Spanish version showed fit statistics in line with standard recommendations: CFI = 0.97; NNFI = 0.97; RMSEA = 0.066; SRMS = 0.077. The internal consistency reliability of the CEQ-E was good for the overall scale (0.88) and for all subscales (0.80, 0.90, 0.76, 0.68 for “own capacity”, “professional support”, “perceived safety” and “participation”, respectively) and similar to the original version. Women with a labour duration ≤ 12 h, women with a labour not induced, women with a normal birth and multiparous women showed higher overall CEQ-E scores and “perceived safety” subscale scores. Women with a labour duration ≤ 12 h and those with previous experience of labour obtained higher scores for the “own capacity” and “participation” subscales.

**Conclusions:**

The results of this study indicate that the CEQ-E can be considered a valid and reliable measure of women’s perceptions of labour and birth in Spain.

**Electronic supplementary material:**

The online version of this article (doi:10.1186/s12884-016-1100-z) contains supplementary material, which is available to authorized users.

## Background

Reproductive health services have traditionally focused its efforts and resources on lowering perinatal mortality. Meanwhile, less attention has been paid to mothers’ individual experiences and beliefs regarding maternity and the childbearing process [1–3].

Globally, maternity care has been structured in different forms and procedures of care, it has been organized over time due to historical and cultural influences [2, 4, 5]. Even though, in Europe, an effort has been made in order to shift it towards a woman-centred care while maintain quality of care [6, 7]. For instance, in Spain an effort has been made in order to deliver evidence-based as well as patient-centred care in the maternity settings [3].

A healthcare system can be improved and developed by involving patients in their own care [3, 8–12]. It has been argued that patient satisfaction and experience of care is an important indicator of quality [9, 13, 14]. Furthermore, it can successfully be used as a predictor of outcomes. The obstetric literature is lacking in women’s psychological health studies, and there is a paucity of patient reported outcomes (PRO) when evaluating patient experience [15]. It has been openly acknowledged that efforts should be made to cover the gap in this area of obstetric care, efforts should be focused into promote a health care based on the patient needs and believes [11, 16]. A negative childbirth experience has been linked to a lower rates of breastfeeding, increase disorders of mother to infant bonding, increased post-partum depression and post-traumatic stress syndrome [17, 18]. Such disorders have been linked to influence the decision, expectations for subsequent pregnancies and the choice of delivery [19, 20]. Furthermore, asking about mothers’ experiences may help to identify maternal needs as well as areas of care in need of improvement. Therefore, more research is needed in this area in order to provide reliable findings on how an intervention may enhance women’s experience of labour and birth [21–23].

In order to evaluate different dimensions of birth experiences, we selected a psychometric approach implemented by means of a postpartum questionnaire that explored women’s experience of childbirth [24]. They have been described as unique tools, providing a standardised analysis as well as a meaningful validation when comparing results within same or different studies [8, 10, 25].

The Childbirth Experience Questionnaire (CEQ), developed by Dencker et al. 2010, was originally designed to study women’s perceptions of their first labour and birth. There is evidence that the Swedish version of the CEQ is a sufficiently reliable and valid tool to evaluate multidimensional postpartum aspects, including women’s perceptions and feelings about their first labour and birth [24]. We selected the CEQ because it is the most recently published multidimensional instrument and the only one that comprehensively evaluates patients’ perception and feelings. Even though it has been transculturally validated for use in the United Kingdom, it has not yet been adapted to the Spanish speaking population [26]. The purpose of this study, therefore, was to adapt the CEQ to the Spanish context and to assess its psychometric properties among Spanish speaking women.

## Methods

### Aim

The aim of the study was to adapt the Childbirth Experience Questionnaire (CEQ) to the Spanish context and to determine its psychometric properties.

### Participants

An initial convenience sample of 364 women was recruited from 3 different hospitals in Elda, Elche and Alcoi (eastern Spain) in 2011. All participants were aged over 18 years old and were able to read and write in Spanish. They were admitted to a maternity ward after having a normal or instrumental vaginal delivery. As per original validation study protocol [24], uncomplicated pregnancies: mothers had had singleton term pregnancies (between 37 and 42 weeks of gestation) in a cephalic presentation were included in the study. Furthermore, those with multiple pregnancies, severe maternal or neonatal pathology and caesarean section were excluded from our sample.

### Instrument

The CEQ questionnaire includes 22 items that originally referred to the first childbirth experience. Responses to 19 of the items are scored on a 4-point Likert scale and three of the items are assessed using a visual analogue scale (VAS). The VAS-scale scores are transformed to categorical values; 0–40 = 1, 41–60 = 2, 61–80 = 3 and 81–100 = 4. Negatively worded item scores are reversed. Items are grouped into 4 domains: “own capacity” (8 items regarding sense of control, personal feelings during childbirth and labour pain), “professional support” (5 items about information and midwifery care), “perceived safety” (6 items regarding sense of security and memories from the childbirth), and “participation” (3 items regarding own possibilities to influence position, movements and pain relief during labour and birth). The questionnaire has shown good reliability and construct validity when evaluating childbirth experience between known-groups [24]. The method of known-groups validation was used to assess whether the CEQ can discriminate between groups known to differ on key variables. A recent adaptation of the CEQ questionnaire to the UK population yielded a good test-retest reliability and showed significant differences between known-groups [26]. Furthermore, Walker et al. found a positive strong correlation with the total score of the “Care During Labour and Birth” domain of the 2010 UK Maternity Survey, a tool used as a gold standard for measuring the quality of maternity services in the UK, as an additional evidence for criterion validity of the CEQ.

### Translation procedure

A linguistic validation process was used [27]. First, a translation was carried out from Swedish to Spanish by a bilingual translator, who was asked to grade the difficulty of the translation (1 = not at all difficult; 10 = maximum difficulty) and to classify the type of changes made to each item: 1 = no changes were necessary; 2 = modifications had to be made during translation in order to maintain semantic and conceptual equivalence; and 3 = the item was culturally inappropriate. Another bilingual translator, totally blinded to the original Swedish version, translated the first Spanish version back into Swedish. Each item from this forward-backward translation was contrasted with the original one, and all differences were reported. When the terminology used in the questionnaire required clarification (i.e. differences between delivery and birth), the first author of the original Swedish questionnaire was consulted. Translators and researchers finally agreed on a Spanish version of the CEQ (CEQ-E). Cognitive interviews were conducted with 26 postpartum women to test comprehensibility and legibility of the final CEQ-E [28].

### Data collection

At discharge and after informed consent was obtained, participants completed a questionnaire on sociodemographic variables and confirmed their contact address. We obtained data from maternity records regarding parity, type of onset of labour, birthing time, type of birth (spontaneous vaginal or instrumental) and neonatal intensive care unit admission. The CEQ-E was sent via post between one and 3 months postpartum. If questionnaires were not returned, monthly reminders were sent twice. Questionnaires were posted from the University of Alicante and returned using prepaid postage to the University.

### Data analysis

The planned sample size was 220 women. This was based on a recommended sample size of ten times the number of observed variables [28], with at least five to seven times the number of observed variables in the health measurement tool being evaluated [29]. As recommended by Terwee et al., we also took missing items into account. The results of the CEQ-E were scored as per original validation study [24].

In order to examine the CEQ-E structure, we performed a confirmatory factor analysis (CFA) of polychoric correlations using a diagonally weighted least squares estimator. We tested 3 models: model 1 examined a one factor model, in which the 22 items were assumed to be indicators of a single latent factor. Model 2 assessed the presence of 4 related latent variables, according to the conceptual structure of the original study. The items included in each factor of model 2 were: “own capacity” (items 1–2, 4–6, 19–21), “professional support” (items 13–17), “perceived safety” (items 3, 7–9, 18, 22), and “participation” (items 10–12). Model 3 evaluated the original structure changing the location of item 18, consistent with its content, from the “perceived safety” to “professional support” domain.

CEQ-E reliability was assessed using the Cronbach’s alpha coefficient. Further testing of construct validity was conducted by testing differences in CEQ-E scores between subgroups known to differ in key variables as per original study [24]. Nevertheless, multiparous women were included in our study analysis, hypothesis yet to be explored. Therefore, based on previous research, it was hypothesised that women with longer labour [24, 26, 30], with induced labour [31], instrumental delivery [24, 26, 31, 32] and women without previous experience of birth [20, 23, 33, 34] would obtain lower scores on the questionnaire.

The Mann–Whitney *U* test was used to contrast hypotheses. Effect sizes, as defined by Cohen [35], were computed as the difference between group mean scores divided by the pooled standard deviation of the two groups. As suggested, effect sizes of 0.2–0.5 were regarded as “small”, 0.5–0.8 as “moderate” and above 0.8 as “large” [28]. Data were analysed using SPSS 15.0 for Windows and LISREL 8.8.

## Results

### Description of sample and test information

Of the 364 eligible women that met the inclusion criteria, 226 (62.1%) completed and returned the CEQ-E questionnaire between 1 and 3 months postpartum. Other characteristics can be seen in Table 1. The Table 2 shows the proportion of extreme-value responses and the means and standard deviations of the CEQ-E item responses.

**Table 1:** Characteristics of the study population *n* = 226

Variables	N (1–3 m)	%
Country of birth		
Spain	212	93.8
Other	14	6.2
Education		
High school or below	95	42.6
College or above	128	57.4
Marital status		
Married/registered partnership	196	87.9
Separated/divorced/widowed/single mother	27	12.1
Maternal age, years, mean (SD)	31 (5)	
Gestational age weeks, mean (SD)	39.3 (1.3)	
Previous deliveries		
Yes	132	58.4
No	94	41.6
Onset of labour		
Spontaneous	88	38.9
Induction	51	22.6
Labour duration more than 12 h	59	26.2
Type of delivery		
Spontaneous vaginal	182	80.5
Instrumental	43	19.0
Perineal status after birth		
Tear	53	23.7
Episiotomy	148	66.1
Neonatal Intensive Care Unit Admission (only for observation)	8	3.7
Birth Hospital		
Elche	129	57.1
Alcoi	52	23
Elda	45	19.9

**Table 2:** Childbirth experience questionnaire (CEQ) item description^a^

Item	Total sample per item (*n* = 226)	Floor %^b^ ‘Totally disagree’	Ceiling %^b^ ‘Totally agree’	M	SD
1. The labour progress went as I has expected	226	11.9	18.1	2.72	0.90
2. I felt strong	226	2.2	30.5	3.12	0.72
3. I felt scared^R^	226	13.7	21.2	2.58	0.97
4. I felt capable	226	2.7	35.0	3.14	0.77
5. I felt tired^R^	226	20.8	11.1	2.26	0.91
6. I felt happy	226	5.3	44.7	3.18	0.89
7. I have many positive memories from the labour process	226	3.5	44.2	3.24	0.81
8. I have many negative memories from the labour prices^R^	226	7.1	37.6	3.07	0.91
9. Some of my memories from the labour process make me feel depressed^R^	226	6.6	54.4	3.30	0.91
10. I felt I could choose whether I should be up and moving or lie down	225	10.7	51.6	3.19	1.01
11. I felt I could choose the delivery position	222	19.8	30.2	2.61	1.11
12. I felt I could choose which pain relief method to use	225	14.7	34.7	2.85	1.06
13. My midwife devoted enough time to me	226	1.3	70.8	3.66	0.58
14. My midwife also devoted enough time to my partner	226	2.2	59.7	3.49	0.71
15. My midwife kept me informed about what was happening during labour and birth	226	1.3	67.7	3.58	0.68
16. My midwife understood my needs	225	1.8	63.6	3.55	0.67
17. I felt very well taken care of by the midwife	226	1.8	74.8	3.69	0.60
18. My impression of the medical competence made me feel secure	226	0.4	68.6	3.66	0.53
19. I felt that I handled the situation well	225	0.4	29.8	3.14	0.67
20. Experienced level of labour pain, VAS^a^ ^R^	226	27.9	20.8	2.31	1.09
21. Experienced level of control, VAS^a^	226	25.2	19.0	2.42	1.06
22. Experienced level of sense of security; VAS^a^	225	15.1	32.4	2.75	1.07

^R^ratings of negatively worded statements are reversed

^a^VAS-scales scores were recoded to categorical values, 0–40 = 1, 41–60 = 2, 61–80 = 3 and 81–100 = 4

^b^% of the top (ceiling) or the bottom (floor) responses per item are shown

Reproduced with authors’ permission. Use of the survey granted © 2010 Dencker et al.; licensee BioMed Central Ltd

### Semantic equivalence

We did not find any semantic differences, so there were no real differences in meaning, but we did find some syntactic or stylistic changes in the Spanish version because conventions are different or because of the context. In addition, in the cognitive interviews, we did not find any disagreement that required the modification of an item.

All the changes made were modifications introduced during the translation process in order to maintain semantic and conceptual equivalence (type 2 as classified above). Sentences were added in the instructions in order to adapt the instrument to our healthcare context (“in the maternal-infant area”) and ensure courtesy (“please”). For item 9, we changed the word “depressed” to “sad”, since the original term has much stronger negative connotations in Spanish. In item 11, we explained the term “birth position” in Spanish (“position that I must assume in order to push my baby out”) in order to use better, less medical language. In item 12, we chose “pain relief methods” instead of “painkiller methods”, since this latter expression is associated with “tablets or pills” in Spanish. In items 13 and 14, we changed “time” to “attention”, since this word in Spanish implies not only time but also observant care. In item 18, “medical competence” was changed to “professional competence”, since “medical competence” does not make much sense in Spanish. Finally, for items 20, 21 and 22, the syntactic form of the sentence was changed to improve question clarity.

### Factor analysis

Table 3 shows the fit statistics of confirmatory factor analyses. The original structure solution presented an insufficient fit index; however, fit was significantly improved by changing the location of one item (item 18) from the “perceived safety” domain to “professional support”. Therefore, model 3 was used as the final CEQ-E version.

**Table 3:** Confirmatory factor analyses: fit statistics

Models	CFI	NNFI (TLI)	RMSEA	SRMR	Satorra-Bentler χ^2^	df
1 factor (model 1)	.76	.74	.200	.160	2092	209
4 factors (model 2)	.97	.96	.075	.100	462.7	203
4 factors (model 3)	.97	.97	.066	.077	402.7	203
Standard cut-off values	>.95	> .95	< .06	< .08		

*CFI* comparative fit index, *NNFI* (*TLI*) non normed fit index (tucker lewis index), *RMSEA* root mean square error of approximation, *SRMR* standardized root mean square residual

### Internal consistency

Internal consistency of the CEQ-E was 0.80, 0.90, 0.76 and 0.68 for “own capacity”, “professional support”, “perceived safety” and “participation” respectively. Internal consistency of the two subscales with a different set of items when the original structure was considered were: 0.88 and 0.75 for “professional support” and “perceived safety” respectively. The overall Cronbach’s alpha was 0.88.

### Known-group validation

Known-group validation was used to assess construct validity (see Table 4). Women whose duration of labour was shorter than 12 h obtained significantly higher scores in each of the following subscales: “own capacity”, “perceived safety” and “participation”, and an overall higher CEQ-E score than women with longer labour. Women with spontaneous onset of labour and women with spontaneous vaginal birth obtained significantly higher scores for the “perceived safety” subscale and the overall CEQ-E score, when compared to women with induced labour and instrumental delivery, respectively. Multiparous women obtained higher scores than nulliparous ones for “own capacity”, “perceived safety” and “participation” subscales, as well as for the overall CEQ-E score. As shown in Table 4, no statistically significant differences were found for any of the hypothesised groups for the “professional support” subscale.

**Table 4:** Differences in subscale scores and overall score by different groups

	Own Capacity^c^	Professional Support (CEQ-E)^a^	Professional Support (Original)^b^	Perceived Safety (CEQ-E) ^a^	Perceived Safety (Original)^b^	Participation^c^	Mean CEQ-E Score^a^	Mean CEQ Score^b^
Labour duration ≤12 h, *n* = 166	2.86 (0.56)	3.60 (0.52)	3.59 (0.55)	3.04 (0.67)	3.14 (0.59)	2.96 (0.83)	3.12 (0.49)	3.14 (0.48)
Labour duration >12 h, *n* = 59	2.58 (0.55)	3.61 (0.47)	3.61 (0.50)	2.84 (0.67)	2.97 (0.58)	2.69 (0.81)	2.93 (0.44)	2.97 (0.43)
*p* value	0.002	0.906	0.939	0.035	0.044	0.021	0.008	0.009
Cohen’s effect size	0.50	0.02	0.04	0.30	0.29	0.33	0.41	0.37
Spontaneous onset of labour *n* = 175	2.82 (0.57)	3.62 (0.51)	3.60 (0.53)	3.04 (0.66)	3.15 (0.59)	2.95 (0.82)	3.11 (0.48)	3.14 (0.48)
Induced labour *n* = 51	2.66 (0.56)	3.58 (0.52)	3.58 (0.56)	2.80 (0.68)	2.93 (0.57)	2.69 (0.83)	2.93 (0.45)	2.96 (0.44)
*p* value	0.086	0.54	0.79	0.020	0.015	0.065	0.012	0.017
Cohen’s effect size	0.28	0.08	0.04	0.36	0.38	0.30	0.39	0.39
Spontaneous vaginal birth *n* = 182	2.82 (0.57)	3.61 (0.53)	3.60 (0.55)	3.04 (0.66)	3.14 (0.59)	2.92 (0.82)	3.10 (0.48)	3.13 (0.47)
Instrumental delivery *n* = 43	2.65 (0.57)	3.60 (0.45)	3.56 (0.50)	2.77 (0.68)	2.94 (0.59)	2.71 (0.84)	2.93 (0.48)	2.97 (0.47)
*p* value	0.081	0.60	0.37	0.020	0.049	0.192	0.031	0.035
Cohen’s effect size	0.30	0.02	0.08	0.40	0.34	0.25	0.35	0.34
Primiparous, *n* = 118	2.69 (0.55)	3.60 (0.51)	3.58 (0.54)	2.91 (0.63)	3.04 (0.54)	2.77 (0.85)	2.99 (0.45)	3.02 (0.44)
Multiparous, *n* = 108	2.89 (0.57)	3.62 (0.52)	3.61 (0.54)	3.06 (0.71)	3.17 (0.63)	3.02 (0.79)	3.16 (0.50)	3.18 (0.49)
*p* value	0.007	0.43	0.503	0.043	0.043	0.035	0.007	0.007
Cohen’s effect size	0.36	0.04	0.06	0.22	0.22	0.30	0.36	0.34

Data presented as mean (SD). Total score for the CEQ is the mean score of the 4 subscales. Numbers given to 2 significant figures

^a^The original conceptual structure changing the location of item 18, from the “perceived safety” to “professional support” domain (Model 3) was used to calculate the mean scores

^b^Conceptual structure of the original study (Model 2) was used to calculate the mean scores

^c^The items included in these subscales are the same for conceptual structure of the original study and CEQ-E

## Discussion

This study offers a transcultural adaptation of the Childbirth Experience Questionnaire (CEQ) to the Spanish context. In order to implement and improve maternity health services, mothers’ views and expectations should be taken into account [6, 22, 36]. Questionnaires such as the CEQ provide the opportunity to study mothers’ experiences of labour care in order to tailor women’s care to their needs and circumstances. The Spanish version of the CEQ has shown similar reliability and validity to the original Swedish and adapted English versions [24, 26]. Despite using a postal questionnaire, we achieved a final sample size of 226 women, with a response rate of 61.3%, meeting the minimum recommended sample size [28, 29].

The translation process was systematically and rigorously conducted to ensure that equivalence was established. It was difficult to find a fluent Swedish-Spanish translator knowledgeable in maternity care, and consequently only two translators were used. Therefore, only one forward and one backward translation were carried out. However, to ensure an equivalent version, the translation process was reviewed by a committee which included the author of the original questionnaire.

We performed a confirmatory factor analysis (CFA) based on the original four-dimension factor structure. Although the CFA of the original structure indicated marginal fit statistics, the proposed Spanish version, in which item 18 was moved from the “perceived safety” to the “professional support” dimension, showed fit statistics in line with standard recommendations [29]. The dimension of this item was changed in coherence with its related content. The internal consistency of the CEQ-E was good and similar to the original and the revised UK version for each of the domains [24, 26].

When the questionnaire was tested via known-group validation, Dencker et al. found statistically significant differences when comparing women with labour lasting less than 12 h, women with no oxytocin augmentation during labour, and spontaneous vaginal birth, versus women with longer labour, oxytocin augmentation and instrumental delivery, respectively, for all subscales [24]. In our study, known-group results offered validity of the CEQ-E in the same direction as for the original study [24] and similar to the English adaptation [26].

The differences observed in statistical significance and magnitude of effect size between the original CEQ study and ours may be a result of operationalisation of the variables studied as well as the different contexts in which the studies were conducted. The first CEQ study was based on a previous prospective randomised study as described elsewhere [24], whereas ours was an observational study with a less precise operationalisation of variables. For example, labour duration was originally measured from the moment when cervical dilatation was at least 4 cm, whereas in our study labour was measured from women’s admission to the delivery suite when regular contractions were reported, spontaneous premature rupture of membranes occurred or drugs were administered for augmentation or induction of labour, regardless of cervical dilatation. Hence, it should be assumed that most women were probably not in the active phase of labour at the time of admission in our study, reducing differences in our sample when comparing the CEQ-E scores by labour duration.

Furthermore, it should be noted that oxytocin infusion is routinely used in Spanish labour wards for the majority of women in labour. In our study, we therefore decided to use the onset of labour variable instead of the one proposed in the original study: oxytocin augmentation during labour. The fact that many women with spontaneous onset of labour would routinely receive oxytocin infusion during childbirth could explain the smaller differences between the CEQ-E scores when compared with mothers with induced labour.

In addition, even though the Swedish study included emergency caesarean section delivery (30% of operative deliveries were caesarean section) as mode of instrumental delivery, we did not include intrapartum emergency caesarean. It has previously been argued that surgical deliveries negatively affect childbirth experiences more than any other operational vaginal birth [33, 37]. This may also have reduced CEQ score differences regarding types of delivery in our study.

As for the context in which the studies were conducted, one explanation for differences in the CEQ scores could be that the care offered in Spain and Sweden is different, as shown by the latest perinatal health reports. These differences are evident when comparing the episiotomy ratio (58% in Spain versus 6.6% in Sweden) or induction of labour rates (31.7% as against 13.7%) [38, 39]. According to the hypotheses proposed excessive interventionism could reduce scores of the CEQ [23], which renders an accurate comparison between studies more difficult.

In contrast to the original Swedish study, we did not observe any significant differences in the “professional support” subscale for any of the hypotheses proposed for the known-group validation method. Walker et al. obtained similar results to ours in their transcultural adaptation of the questionnaire to the UK population [24, 26]. The 5 items in the “professional support” domain showed a high ceiling effect, reducing their sensitivity to differences between comparison groups.

As a novelty, in our study, we included multiparous women in our sample and these were compared with primiparous women when construct validity was tested. As an additional test for construct validity, we have proven that the Spanish version of the CEQ questionnaire discriminates between primiparous and multiparous women’s birth experiences. Even though this relationship has not been fully clarified yet, some authors have stated that women with previous birth experiences were more likely to have a positive experience than first time mothers [23, 33, 34]. In our study, as it was hypothesised, multiparous women obtained higher scores, thus indicating a more positive birth experience.

The main limitation of this study, as previously mentioned, was that it would have been desirable to have had more accurate records regarding the use of intrapartum oxytocin infusion, the inclusion of caesarean section during labour, high risk pregnancies, preterm deliveries as well as the precise timing for onset of labour. Another limiting factor could be discussed due to the methods used for data collection. In some questionnaires a recall of 5 months could be seen after a twice monthly reminders were sent. As some authors have argued, time lapses between birth and study survey could possibly negatively influence on experience [40–42].

## Conclusion

The present study includes the translation and psychometric validation (reliability, psychometric validation, confirmatory factorial structure and known-group validation) of a Spanish version of the CEQ (CEQ-E). Data from this study demonstrate that the Childbirth Experience Questionnaire is a valid and reliable measure of childbirth experience in the Spanish population. In addition, this paper supports the use of the CEQ with multiparous women, opening the door to measuring different aspects of labour and birth regardless of parity.

## Additional file

Additional file 1: The Childbirth Experience Questionnaire – CEQ (Spanish version). Spanish adapted version of The Childbirth Experience Questionnaire. (DOC 105 kb)

## Abbreviations

CEQ: Childbirth experience questionnaire
CEQ-E: Childbirth experience questionnaire Spanish version
CFA: Confirmatory factor analysis
UK: United Kingdom
VAS: Visual analogue scale
