# Supplementary material for: The Spanish version of the Childbirth Experience Questionnaire (CEQ-E): reliability and validity assessment
Source: BMC Pregnancy Childbirth. 2016 Nov 24;16:372. doi: 10.1186/s12884-016-1100-z (PMC5123212; doi:10.1186/s12884-016-1100-z)
Supplement: Additional file 1: — The Childbirth Experience Questionnaire – CEQ (Spanish version). Spanish adapted version of The Childbirth Experience Questionnaire. (DOC 105 kb) [file 12884_2016_1100_MOESM1_ESM.doc]

**Cuestionario sobre tus experiencias durante el parto**

¡Para ti que acabas de ser madre!

Uno de los objetivos de los profesionales es, en el área materno-infantil de tu departamento de salud, el de ofrecer una experiencia positiva durante el parto. Así pues, el propósito de este cuestionario es conocer tus experiencias durante el parto, y comparar tus respuestas con las de otras madres, para poder evaluar la atención que ofrecemos. Por favor, es importante que respondas todas las cuestiones.

Hay respuestas en las que debes marcar con una cruz la casilla y respuestas en las que debes marcar con una cruz una línea:

Ejemplo 1. Lee la frase y marca con una cruz la casilla que mejor describa tu respuesta.

Como fruta cada día.

| Totalmente  de acuerdo | Bastante  de acuerdo | Bastante  en desacuerdo | Totalmente  en desacuerdo |
| --- | --- | --- | --- |
| □ | ⮽ | □ | □ |

Ejemplo 2. Lee la frase y marca con una cruz el tramo de línea que mejor describa tu respuesta.

¿Te gustan las manzanas?

X

Nada, Son lo mejor

en absoluto que hay

*El cuestionario empieza en la próxima página.*

*¡Gracias por darnos tu punto de vista y por colaborar con tus respuestas!*

1. **El parto fue como esperaba.**

| Totalmente  de acuerdo | Bastante  de acuerdo | Bastante  en desacuerdo | Totalmente  en desacuerdo |
| --- | --- | --- | --- |
| □ | □ | □ | □ |

1. **Me sentí fuerte durante el parto.**

| Totalmente  de acuerdo | Bastante  de acuerdo | Bastante  en desacuerdo | Totalmente  en desacuerdo |
| --- | --- | --- | --- |
| □ | □ | □ | □ |

1. **Tenía miedo durante el parto.**

| Totalmente  de acuerdo | Bastante  de acuerdo | Bastante  en desacuerdo | Totalmente  en desacuerdo |
| --- | --- | --- | --- |
| □ | □ | □ | □ |

1. **Me sentí capaz durante el parto.**

| Totalmente  de acuerdo | Bastante  de acuerdo | Bastante  en desacuerdo | Totalmente  en desacuerdo |
| --- | --- | --- | --- |
| □ | □ | □ | □ |

1. **Me sentí cansada durante el parto.**

| Totalmente  de acuerdo | Bastante  de acuerdo | Bastante  en desacuerdo | Totalmente  en desacuerdo |
| --- | --- | --- | --- |
| □ | □ | □ | □ |

1. **Me sentí feliz durante el parto.**

| Totalmente  de acuerdo | Bastante  de acuerdo | Bastante  en desacuerdo | Totalmente  en desacuerdo |
| --- | --- | --- | --- |
| □ | □ | □ | □ |

1. **Tengo muchos recuerdos positivos del parto.**

| Totalmente  de acuerdo | Bastante  de acuerdo | Bastante  en desacuerdo | Totalmente  en desacuerdo |
| --- | --- | --- | --- |
| □ | □ | □ | □ |

1. **Tengo muchos recuerdos negativos del parto.**

| Totalmente  de acuerdo | Bastante  de acuerdo | Bastante  en desacuerdo | Totalmente  en desacuerdo |
| --- | --- | --- | --- |
| □ | □ | □ | □ |

1. **Me ponen triste algunos recuerdos del parto.**

| Totalmente  de acuerdo | Bastante  de acuerdo | Bastante  en desacuerdo | Totalmente  en desacuerdo |
| --- | --- | --- | --- |
| □ | □ | □ | □ |

1. **Me pareció que podía elegir entre estar levantada y moviéndome o estar acostada.**

| Totalmente  de acuerdo | Bastante  de acuerdo | Bastante  en desacuerdo | Totalmente  en desacuerdo |
| --- | --- | --- | --- |
| □ | □ | □ | □ |

1. **Me pareció que podía elegir la posición cuando tuve que empujar para que saliera el bebé.**

| Totalmente  de acuerdo | Bastante  de acuerdo | Bastante  en desacuerdo | Totalmente  en desacuerdo |
| --- | --- | --- | --- |
| □ | □ | □ | □ |

1. **Me pareció que podía elegir entre diferentes métodos para calmar el dolor.**

| Totalmente  de acuerdo | Bastante  de acuerdo | Bastante  en desacuerdo | Totalmente  en desacuerdo |
| --- | --- | --- | --- |
| □ | □ | □ | □ |

1. **La matrona me dedicó la atención necesaria.**

| Totalmente  de acuerdo | Bastante  de acuerdo | Bastante  en desacuerdo | Totalmente  en desacuerdo |
| --- | --- | --- | --- |
| □ | □ | □ | □ |

1. **La matrona le dedicó la atención necesaria a mi pareja.**

| Totalmente  de acuerdo | Bastante  de acuerdo | Bastante  en desacuerdo | Totalmente  en desacuerdo |
| --- | --- | --- | --- |
| □ | □ | □ | □ |

1. **La matrona me mantuvo informada sobre lo que estaba pasando durante el parto.**

| Totalmente  de acuerdo | Bastante  de acuerdo | Bastante  en desacuerdo | Totalmente  en desacuerdo |
| --- | --- | --- | --- |
| □ | □ | □ | □ |

1. **La matrona entendió mis necesidades.**

| Totalmente  de acuerdo | Bastante  de acuerdo | Bastante  en desacuerdo | Totalmente  en desacuerdo |
| --- | --- | --- | --- |
| □ | □ | □ | □ |

1. **Me sentí muy bien atendida por la matrona.**

| Totalmente  de acuerdo | Bastante  de acuerdo | Bastante  en desacuerdo | Totalmente  en desacuerdo |
| --- | --- | --- | --- |
| □ | □ | □ | □ |

1. **Me dio seguridad la competencia de los profesionales.**

| Totalmente  de acuerdo | Bastante  de acuerdo | Bastante  en desacuerdo | Totalmente  en desacuerdo |
| --- | --- | --- | --- |
| □ | □ | □ | □ |

1. **Me sentí capaz de manejar bien la situación.**

| Totalmente  de acuerdo | Bastante  de acuerdo | Bastante  en desacuerdo | Totalmente  en desacuerdo |
| --- | --- | --- | --- |
| □ | □ | □ | □ |

1. **En general, durante el parto, ¿sentiste dolor?**

Ningún dolor El peor dolor

imaginable

1. **En general, durante el parto, ¿sentiste que tenías control?**

Nada de control El máximo control

posible

1. **En general, durante el parto, ¿te sentiste segura?**

No me sentí nada Me sentí segura

segura del todo

**Escribe aquí otros comentarios que quieras hacer:**

¡Muchas gracias por tu colaboración!
